# Effect of interesting respiratory rehabilitation training for the treatment of refractory *Mycoplasma pneumoniae* pneumonia in children

**DOI:** 10.1186/s12879-023-08513-4

**Published:** 2023-08-28

**Authors:** Wenqing Li, Ting Liu, Min Yao, Ru Yu, Meiyan Shu, Maorong Zhang, Jing Huang

**Affiliations:** Department of Respiratory Medicine, Wuhan Children’s Hospital, Tongji Medical College, Huazhong University of Science and Technology, Wuhan, China

**Keywords:** Respiratory rehabilitation training, Refractory *Mycoplasma pneumoniae* pneumonia, Pulmonary function, Interesting training

## Abstract

**Backgrounds:**

Refractory *Mycoplasma pneumoniae* pneumonia (RMPP) cause damage of pulmonary function and physical therapy assisting medical treatment is needed.

**Objective:**

The aim of this study was to investigate the effect of interesting respiratory rehabilitation training on pulmonary function in children with RMPP.

**Methods:**

A total of 76 children with diagnoses of RMPP in our hospital from January 2020 to February 2021 were enrolled in this prospective study. According to the random number table method, they were divided into the control group and the study group, with 38 cases in each group. The control group were given conventional treatment, and the study group received interesting respiratory rehabilitation training in the basis of conventional treatment. The antipyretic time, disappearance time of pulmonary shadow and cough, length of hospital stay, pulmonary function (first second of expiratory volume (FEV1), forced vital capacity (FVC), FEV1/FVC) at 1 day before and after intervention, serum interleukin-6 (IL-6), C-reactive protein (CRP), tumor necrosis factor (TNF-α), and quality of life (Pediatric Quality of Life Inventory, PedsQL 4.0 scale) were observed in the two groups.

**Results:**

The antipyretic time, disappearance time of pulmonary shadow and cough, length of hospital stay in the study group were shorter than those in the control group (*P* < 0.05). One day before intervention, there was no significant difference in FVC, FEV1, FEV1/FVC IL-6, CRP, and TNF-α between the two groups (*P* > 0.05). One day after intervention, FVC, FEV1 and FEV1/FVC in the study group were better than those in the control group (*P* < 0.05), and the levels of IL-6, CRP, and TNF-α in the study group were lower than those in the control group with significant difference (*P* < 0.05). One day before intervention, there were no significant differences in physiological function, emotional function, social function, and school function between the two groups (*P* > 0.05). After intervention, physiological function, emotional function, social function, and school function of the study group were better than those of the control group (*P* < 0.05).

**Conclusion:**

The interesting respiratory rehabilitation training can effectively improve the pulmonary function of children with RMPP, with strong flexibility, which is worthy of clinical application.

## Introduction

*Mycoplasma pneumoniae* pneumonia (MPP) is a common pneumonia in school-age children and adolescents, mainly caused by *mycoplasma pneumoniae* infection, which can occur throughout the year, accounting for 10-40% of community-acquired pneumonia in hospitalized children [1, 2]. The most common clinical manifestations of MPP include dry cough and fever, often accompanied by headache, myalgia, and sore throat, as well as support for auxiliary department examinations (elevated inflammatory markers) and abnormal imaging findings [3, 4]. The clinical symptoms of the disease are similar to influenza, and misdiagnosis is easy. Some children may thus progress to refractory MPP (RMPP).

The definition of RMPP is as follows: the fever in children with MPP infection last for more than 10 days and the c-reactive protein level is over 40 mg/L. Additionally, there is a high density uniform solid window and the condition aggravate though using routine macrolide antibiotics for more than seven days [5]. The pulmonary dysfunction in children with RMPP is mainly mixed ventilation dysfunction, obstructive ventilation dysfunction and restrictive ventilation dysfunction, among which the most common is mixed ventilation dysfunction. Additionally, there may be small and large airway injuries. In addition to severe lung lesions, RMPP often involves multiple systems outside the lung, including nervous system, cardiovascular system, gastrointestinal tract, kidney, blood system, bone, joint, muscle and skin.

Since the incidence of RMPP is increasing year by year with long disease course, although the symptoms can be relieved after treatment, the pulmonary function of children is accompanied by different degrees of damage, which needs to be repaired. Moreover, there is a certain difficulty in treatment, which has been the focus of clinical research. To avoid drug abuse, it is critical to search for an appropriate intervention point for initiating steroid treatment. At present, expectorant drugs and macrolide antibiotics are often used to treat young patients with RMPP for at least 7 days. Some children will have adverse reactions such as hormone resistance, restlessness and gastrointestinal reactions, which will affect the clinical outcomes [6]. What’s more, too late corticosteroid intervention may lead to sustained inflammatory damage or even irreversible organ damage [7]. Therefore, it is important for searching an economical, safe and side-effect-free physical therapy to assist conventional treatment of RMPP.

Chest physiotherapy is an important adjuvant in the treatment of most respiratory illnesses [8]. The purpose or benefits of chest physiotherapy include evacuating inflammatory exudates and tracheobronchial secretions, removing airway obstructions, decreasing airway resistance, improving air exchange and diminishing work of breathing [9, 10]. Pulmonary rehabilitation has established itself as a fundamental component in the chest physiotherapy. Considering that breathing training is difficult for children to operate and maintain, we consider adding some funny games into the training mode, which can effectively improve their lung function, divert their attention, improve their treatment compliance, and have a positive impact on the disease. However, there were few studies focused on the correlation of interesting respiratory rehabilitation training with the outcomes of children with pneumonia. Based on this, this study will explore the effect of interesting respiratory rehabilitation training on pulmonary function in children with RMPP.

## Patients and methods

### Clinical data

According to the formula n = 2[(u_1_-α + u_1_-β) s/σ]^2^, α = 0.05, β = 0.01, n = 65 after calculating. Based on the provisions of the State Food and Drug Administration, 15% is the shedding release rate, so the sample size of the group is determined to be n = 65 × 1/(1-0.15) = 75.92 ≈ 76. Therefore, 76 children with RMPP admitted to our hospital from January 2020 to February 2021 were selected as the study subjects. According to the random number table method, they were divided into control group and study group, with 38 cases in each group. The study protocol was approved by the Ethics Committee of Wuhan Children’s Hospital, Tongji Medical College, Huazhong University of Science and Technology. The study protocol complies with the relevant requirements of the Declaration of Helsinki. All patients’ families were informed and signed the consents.

### Inclusion and exclusion criteria

Inclusion criteria: (1) meet the diagnostic criteria for children with refractory *Mycoplasma pneumoniae* pneumonia in the Expert Consensus on the Diagnosis and Treatment of *Mycoplasma pneumoniae* Pneumonia in Children (2015 edition) [11]: after regular treatment with macrolides for 1 week or more, the symptoms of the child did not significantly improve or worsen, the child still had a persistent fever, and the chest imaging findings were worse than before; MP-IgM titer greater than 1:160 or can detect a positive MP-DNA quantitation; (2) without previous history of recurrent respiratory tract infection or chronic pulmonary disease; (3) 5–12 years old; (4) body mass index of 19–23 kg/m^2^.

Exclusion criteria: Patients with (1) disease of immune system; (2) mixed infection with other pathogens; (3) respiratory distress syndrome; (4) other serious underlying diseases or complications; (5) ventilator-assisted ventilation; (6) consciousness disorder and intellectual disability.

### Treatment

The control group was given routine treatment, including 3-day continuous application of azithromycin (10 mg/kg, ivgtt, once a day), and then drug withdrawal for 4 days, as a course of treatment, continuous treatment for 2–3 courses. At the same time, according to the patient’s symptoms, corresponding treatment was given to relieve fever, cough, phlegm, and to protect organ function and maintain water and electrolyte balance [12].

The study group received interesting respiratory rehabilitation training on the basis of conventional treatment used in the children of the control group, specific contents are as follows: (1) Charge nurse and responsible physical therapist(more than N2 level) in respiratory ward were trained for specialized operation training by national rehabilitation specialist nurse, and after theory and skills examination, they could conduct clinical practice guided and supervised by junior nurses and the head nurse. At the same time, the children’s fun game venue was established, decorated with cartoon patterns, balloons, flowers, etc. in walls, and equipped with children’s books, tables and chairs, toys, etc., dressed by spliced foam floor on the ground (2). The physical therapists and responsible nurses will lead the children to the games venues, transfer the children’s interest to the game, and encourage them to pay attention to complete the respiratory training. The physical therapists helped responsible nurses to personalize training plan according to the actual circumstance of the child to, and regularly guided to adjust plans according to the needs of children. The children’s daily training was completed by the physical therapists who has been trained above (3). Before training, the inductive inquiry was performed to collect children’s interests and hobbies. Then the content of interaction was confirmed according to their interests. At the same time, some interesting cards which were popular in the children were taken as prizes for children to collect for every 10 cards for exchange of an interesting prize. If the children’s compliance was not good, the praise cards and prizes were prepared to encourage them to cooperate with the treatment bravely (4). The respiratory function training guidance included lip breathing, abdominal breathing, and three-ball breathing trainer with 8-level and 9-level expiratory conditioning. According to the situation of children, the level was adjusted gradually appropriately for increased resistance training for twice a day, 15–20 min every time. It was appropriate to ensure that children did not feel tired, and they could get a card for successfully completed a training. In addition, in order to improve the children’s training interest, children could blow confetti or newspaper, whistle or recorder, etc. in training spare time. In the interval of treatment, breath training gymnastics should be organized every day, which mainly included standing in place, upper arm lifting, chest expanding exercise, lower limb raising training, etc., with a rest (rest to attain mental composure in music) of 2–3 min per activity and a total time of less than 20 min. The training was performed continuously for one month.

### Observation indicators

The antipyretic time, disappearance time of pulmonary shadow and cough, length of hospital stay were observed and recorded.

Pulmonary function: The children were tested with Maibang MSA99 pulmonary function tester, including first second of expiratory volume (FEV1), forced vital capacity (FVC), FEV1/FVC at 1 day before and 1 day after intervention.

Serum levels of interleukin-6 (IL-6), C-reactive protein (CRP), tumor necrosis factor (TNF-α): 5mL of the venous blood was collected at 1 day before and after intervention and centrifuged for detection. The levels of IL-6 (anhui Joyee Biotechnics Co.,Ltd, JER-04), CRP, TNF-α (Wuhan MSK Biotechnology Co., LTD, kt25328、kt30278) were determined by enzyme-linked immunosorbent assay (ELISA), purchased from Shanghai enzyme-linked biotechnology co., LTD.

Quality of life: The PedsQL [13] (Pediatric Quality of Life Inventory) (Children’s Hospital and Health Center, San Diego, California) is a modular instrument for measuring health-related quality of life (HRQOL) in children and adolescents ages 2 to 18. The health survey questionnaire was distributed at 1 day before and after intervention for evaluation. The scale includes 4 dimensions, consisting of 23 items, physiological function, emotional function, social function, and school function, for 100 points for each dimension. Higher score means higher quality of life.

### Statistical analyses

SPSS 20.0 statistical software was used to analyze the data: the average ± standard deviation (mean ± SD) was used to describe measurement data such as antipyretic time, disappearance time of pulmonary shadow and cough, length of hospital stay. The T test was used for comparison between two groups. If the data did not conform to normal distribution, the median and interquartile M(Q1-Q3) were used for description, and the rank-sum test was used. The frequency (constituent ratio) was used to describe the equal count data like gender. The Wilcoxon test was used to compare the unidirectional ordered data, and the chi-square test was used to compare the disordered data. *P* < 0.05 indicates significant difference.

## Results

### The general information

There were 22 males and 16 females in the study group with the average of 8.3 ± 2.3 years (ranging from 5.2 to 11.8 years). There were 23 males and 15 females in the control group with the average age of 8.2 ± 2.1 years (range, 5.2 to 11.9 years). The general data of the two groups were compared (*P* > 0.05, see Table 1), showing comparability.

**Table 1:** The general information

	Study group (n = 38)	Control group (n = 38)	χ^2^/t	*P*
Gender (male/female)	22/16	23/15	0.054	0.815
Age (year)	8.3 ± 2.3	8.2 ± 2.1	0.362	0.718
Disease course (day)	13.91 ± 2.38	13.85 ± 2.32	0.111	0.912
Fever duration (day)	4.93 ± 0.34	4.91 ± 0.35	0.253	0.801
Cough duration (day)	7.56 ± 0.56	7.52 ± 0.58	0.306	0.761

### The antipyretic time, disappearance time of pulmonary shadow and cough, length of hospital stay between two groups

The antipyretic time, disappearance time of pulmonary shadow and cough, length of hospital stay in the study group were shorter than those in the control group, and the differences were significant (*P* < 0.05), as shown in Table 2.

**Table 2:** The antipyretic time, disappearance time of pulmonary shadow and cough, length of hospital stay between two groups

	Study group (n = 38)	Control group (n = 38)	t	*P*
Disappearance time of cough (day)	6.31 ± 1. 03	10.04 ± 1.02	-15.862	<0.001
Antipyretic time (day)	4.34 ± 0.37	5.78 ± 0.43	-15.648	<0.001
Disappearance time of pulmonary shadow (day)	7.03 ± 0.45	9.21 ± 0.56	-18.706	<0.001
Hospital stay (day)	10.38 ± 1.16	12.38 ± 1.09	-7.745	<0.001

### The comparison of pulmonary function between two groups

One day before intervention, there was no significant difference in FVC, FEV1 and FEV1/FVC between the two groups (*P* > 0.05). One day after intervention, FVC, FEV1 and FEV1/FVC in the study group were higher than those in the control group, and the differences were significant (*P* < 0.05), as shown in Table 3.

**Table 3:** The comparison of pulmonary function between two groups

	Time	Study group (n = 38)	Control group (n = 38)	t	*P*
FVC (%)	1d before intervention	89.93 ± 13.29	89.87 ± 13.11	0.02	0.984
	1d after intervention	107.39 ± 13.17	99.76 ± 13.87	2.459	0.016
FEV1 (%)	1d before intervention	81.67 ± 15.23	81.56 ± 15.19	0.032	0.975
	1d after intervention	96.38 ± 15.56	89.76 ± 14.98	2.679	0.009
FEV1/FVC (%)	1d before intervention	88.27 ± 14.28	88.21 ± 14.21	0.018	0.986
	1d after intervention	100.78 ± 14.09	93.28 ± 14.15	2.315	0.023

FEV1, first second of expiratory volume; FVC, forced vital capacity

### The results of laboratory tests

One day before intervention, there were no significant differences in IL-6, CRP, TNF-α levels between the two groups (*P* > 0.05). One day after intervention, the levels of IL-6, CRP, TNF-α in the study group were lower than those in the control group (*P* < 0.05), as shown in Table 4.

**Table 4:** The results of laboratory tests

	Time	Study group (n = 38)	Control group (n = 38)	t	*P*
IL-6 (pg/mL)	1d before intervention	56.38 ± 4.39	56.32 ± 4.32	0.061	0.952
	1d after intervention	8.98 ± 4.23	16.76 ± 4.19	-8.014	<0.001
CRP (mg/L)	1d before intervention	70.92 ± 6.81	70.89 ± 6.69	0.019	0.985
	1d after intervention	8.02 ± 1.02	15.39 ± 1.06	-30.884	<0.001
TNF-α (ng/L)	1d before intervention	4.49 ± 0.35	4.47 ± 0.36	0.246	0.806
	1d after intervention	2.91 ± 0.32	3.65 ± 0.32	-10.081	<0.001

IL-6, serum interleukin-6; CRP, C-reactive protein; TNF-α, tumor necrosis factor

### The quality of life between two groups

One day before intervention, there were no significant differences in physiological function, emotional function, social function, and school function between the two groups (*P* > 0.05). One day after intervention, the point in each dimension in the study group was higher than those of the control group (*P* < 0.05), as shown in Table 5.

**Table 5:** The quality of life between two groups

	Time	Study group (n = 38)	Control group (n = 38)	t	*P*
Physiological function	1d before intervention	48.19 ± 4.03	48.09 ± 4.05	0.108	0.914
	1d after intervention	73.98 ± 3.98	67.92 ± 4.02	6.604	<0.001
Emotional function	1d before intervention	49.78 ± 5.01	49.83 ± 5.06	-0.043	0.966
	1d after intervention	68.91 ± 4.01	60.82 ± 3.98	8.827	<0.001
Social function	1d before intervention	48.87 ± 5.07	48.81 ± 5.06	0.052	0.959
	1d after intervention	67.67 ± 4.76	54.39 ± 4.81	12.097	<0.001
School function	1d before intervention	48.82 ± 5.06	48.78 ± 5.03	0.035	0.972
	1d after intervention	65.38 ± 4.89	58.93 ± 4.71	5.856	<0.001

## Discussion

Because the clinical symptoms of this disease are similar to those of influenza, misdiagnosis is easy to occur, and some children may progress to RMPP [14]. Patients with RMPP are accompanied by many pathological injuries, including increased mucous secretion, bronchial mucosal erosion, mucosal congestion edema, which could cause the airway to block the traffic of the airway [15]. The previous study [16] pointed out that the formation of bronchial mucous fluid was one of the important obstacles in the treatment of *mycoplasma pneumonia*, and it was common in the refractory pneumonia *mycoplasma pneumonia* with the incidence rate about 60%. In addition, due to the narrow bronchial lumen and poor airway ciliary motor function in children, respiratory clearance capacity is insufficient, and unable to effectively discharge sputum as adults, resulting in obstruction of bronchial endocrine mucus discharge. Moreover, *mycoplasma pneumoniae* infection can induce immune activation of inflammatory cells, release a large number of inflammatory factor, not only cause coagulation fibrinolytic system imbalance leading to local airway mucosa microcirculation, but also directly injury bronchial mucosa, causing airway mucous membrane permeability increased, airway mucosa cilia system dysfunction, and secretion increased and discharge reduced of endocrine mucus. All these factors above are the important reasons for the occurrence of refractory *Mycoplasma pneumoniae* pneumonia [17, 18].

At present, there are few clinical drugs for the treatment of RMPP, mainly antibacterial drugs acting on cell membrane, and macrolide antibiotics such as azithromycin and erythromycin are mainly used for children [19]. Azithromycin, as the second generation of macrolide antibacterial drugs, mainly inhibits RNA and protein synthesis by inhibiting the process of cell transpeptide. Azithromycin has the characteristics of good stability, long half-life, strong antibacterial ability and good compliance, so azithromycin is often used in the treatment of RMPP [20]. However, previous studies [21] have pointed out that the pathogenesis of refractory *mycoplasma pneumoniae* pneumonia is relatively complex, including direct damage of mycoplasma, gene mutations induced by infection, immune response and infection of drug-resistant strains. Therefore, it is the focus of clinical attention to promote effective clearance of respiratory secretions and sputum in children with RMPP, without increasing pain. In this study, we adopt an effective physical therapy as an auxiliary method for the clinical treatment of RMPP.

At present, respiratory function training is one of the widely practical clinical rehabilitation measures in China, which is often used in patients with surgery, inhalation injury and chronic obstructive pulmonary disease. With the help of a series of standardized and systematic effective respiratory training, it can enhance respiratory, and muscle strength and endurance, in order to relieve patients’ dyspnea symptoms, which play an important role in improving pulmonary ventilation function and improving respiratory coordination [22]. However, due to the poor compliance of children during treatment, funny training was considered and adopted on the basis of respiratory rehabilitation training in order to improve the compliance of children. The fun environment and rewards could encourage children to bravely face the disease for their favorite cards and prizes and actively cooperate with respiratory rehabilitation training. According to the results of this study, cough relief time, antifebrile time, lung shadow disappeared time and hospital stay in the study group is less than that in the control group, suggesting this interesting respiratory rehabilitation training can effectively shorten the disease course of RMPP in children. Cheng Shunjiao [23] et al. ‘s research pointed out that the kid-fun nursing intervention can help enhance the treatment cooperation of children with mycoplasma pneumonia, shorten the recovery time of clinical symptoms, and improve the clinical efficacy, which was consistent with our findings.

Pulmonary dysfunction will not only affect the normal respiratory function of children, but also lead to repeated cough and wheezing, which seriously affect the physical and mental health of children. Pulmonary function test can objectively reflect the airway function of patients, and is an objective index of airway pathological changes, disease diagnosis and curative effect evaluation [24, 25]. According to the study of Tao Jing [26] et al., respiratory function training for children with pneumonia can significantly improve their lung function and reduce the occurrence of related complications. At the same time, parents have higher recognition of nursing work, which is worthy of recommendation. Du Xiaoyan [27] pointed out that respiratory function training for children with pneumonia can effectively improve their lung function and reduce the risk of complications. The results of this study showed that one day after intervention, FVC, FEV1 and FEV1/FVC in the study group were higher than those in the control group, suggesting that interesting respiratory rehabilitation training can effectively improve the pulmonary function of children with RMPP and promote the recovery of children.

The immune inflammatory over-reaction is the main cause of aggravation of pneumonia and even extrapulmonary complications. IL-6 is a proinflammatory factor that regulates immune response. TNF-α promotes inflammatory reaction, IL-6 and TNF-α regulate cellular immunity together. If the levels of IL-6 and TNF-α increase, the inflammatory reaction will be aggravated [28]. CRP can effectively reflect the level of inflammation in tissues and cells. Because of its simple detection method, CRP is widely used in clinical practice and can be used as a reliable indicator to evaluate the condition and follow-up of children with RMPP [29]. The results of this study showed that one day after intervention, the levels of IL-6, CRP, and TNF-α in the study group were lower than those in the control group, suggesting that interesting respiratory rehabilitation training can effectively reduce the levels of inflammatory factors in children with RMPP. Interesting respiratory rehabilitation training can exercise the lung function of children with RMMP, maintain the lung ventilation function, thus regulate the immune function, which may reduce the level of inflammatory factors.

In this study, the physiological function, emotional function, social function and school function of the study group were higher than those of the control group, suggesting that interesting respiratory rehabilitation training can effectively improve the quality of life of children with RMPP. In the implementation of children’s interesting respiratory rehabilitation training, cartoon patterns, balloons and flowers can attract the attention of children, relieve children’s bad mood, improve compliance, alleviate related clinical symptoms, and thus improve the quality of life of children. Consistently, Ren et al. founded that the effect of personalized rehabilitation counseling intervention on severe pneumonia children was remarkable, which could effectively improve the treatment compliance, alleviating clinical symptoms and improve the quality of life [30]. Zhao et al. founded that strengthening respiratory function training was helpful to reduce complications in children with pneumonia, improve lung function and quality of life, and increase family satisfaction [31].

### Limitation

However, this study also has some limitations. The sample size of this study is small, and it is also a single-center study, which requires further evaluation by expanding the sample size and multi-center study.

## Conclusion

In conclusion, the interesting respiratory rehabilitation training can effectively improve the pulmonary function of children with RMPP. In addition, it could also improve children’s subjective feeling and therapeutic alliance, which is practical and worthy of clinical application.

## Data Availability

The datasets generated and analyzed during the current study are available from the corresponding author on reasonable request.
